# Prominent muscle involvement in a familial form of mitochondrial disease due to a *COA8* variant

**DOI:** 10.3389/fgene.2023.1278572

**Published:** 2023-11-30

**Authors:** Martina Rimoldi, Francesca Magri, Sara Antognozzi, Michela Ripolone, Sabrina Salani, Daniela Piga, Letizia Bertolasi, Simona Zanotti, Patrizia Ciscato, Francesco Fortunato, Maurizio Moggio, Stefania Corti, Giacomo Pietro Comi, Dario Ronchi

**Affiliations:** ^1^ Neuromuscular and Rare Diseases Unit, Fondazione IRCCS Ca’ Granda Ospedale Maggiore Policlinico, Milan, Italy; ^2^ Neurology Unit, Fondazione IRCCS Ca’ Granda Ospedale Maggiore Policlinico, Milan, Italy; ^3^ Dino Ferrari Center, Department of Pathophysiology and Transplantation, University of Milan, Milan, Italy

**Keywords:** mitochondrial myopathy, cytochrome c oxidase deficiency, COA8, mitochondrial encefalomyopathies, whole exome sequencing

## Abstract

Isolated mitochondrial respiratory chain Complex IV (Cytochrome c Oxidase or COX) deficiency is the second most frequent isolated respiratory chain defect. Causative mutations are mainly identified in structural COX subunits or in proteins involved in the maturation and assembly of the COX holocomplex. We describe an Italian familial case of mitochondrial myopathy due to a variant in the COX assembly factor 8 gene (*COA8*). Patient 1 is a 52-year-old woman who presented generalized epilepsy and retinitis pigmentosa at 10 years of age. From her early adulthood she complained about cramps and myalgia after exercise, and bilateral hearing loss emerged. Last neurological examination (52 years of age) showed bilateral ptosis, muscle weakness, peripheral neuropathy, mild dysarthria and dysphonia, cognitive impairment. Muscle biopsy had shown the presence of ragged-red fibers. Patient 2 (Patient 1’s sister) is a 53-year-old woman presenting fatigability, myalgia, and hearing loss. Neurological examination showed ptosis and muscle weakness. Muscle biopsy displayed a diffuse reduction of COX activity staining and ragged-red fibers. Both sisters presented secondary amenorrhea. After ruling out mtDNA mutations, Whole Exome Sequencing analysis identified the novel homozygous *COA8* defect c.170_173dupGACC, p.(Pro59fs) in the probands. Loss-of-function *COA8* mutations have been associated with cavitating leukoencephalopathy with COX deficiency in 9 reported individuals. Disease course shows an early-onset rapid clinical deterioration, affecting both cognitive and motor functions over months, followed by stabilization and slow improvement over several years. Our findings expand the clinical spectrum of *COA8*-related disease. We confirm the benign course of this rare disorder, highlighting its (intrafamilial) clinical variability.

## 1 Introduction

Mitochondrial disorders (MD) are generally characterized by a wide clinical variability which reflects the heterogenous biochemical and molecular defects underlying clinical symptoms. Indeed, the establishment of a definitive diagnosis can be challenging (Chinnery, 1993).

In affected tissues, the prevalent reduction of the residual activity of Cytochrome c Oxidase (COX, the mitochondrial respiratory chain Complex IV) constitutes the biochemical hallmark of isolated COX deficiency. This condition can be due to molecular defects in mitochondrial and nuclear encoded COX structural subunits or, more frequently, with variants in nuclear genes encoding Complex IV assembly factors. Despite the common biochemical signature, disorders featuring isolated COX deficiency might display heterogenous symptoms with variable age at onset ranging from neonatal forms to adult-onset clinical phenotypes (Rak et al., 2016; Brischigliaro et al., 2019; Brischigliaro and Zeviani, 2021). To date, molecular defects in about 30 genes have been reported as the likely cause of isolated or prevalent COX deficiency (Brischigliaro and Zeviani, 2021).

*COA8* (previously known as *APOPT1*) encodes a 206-amino acid protein whose precise function has not been elucidated yet. COA8 localizes to mitochondrial matrix in proximity of inner mitochondrial membrane where it was initially suspected to stimulate the release of cytochrome c as a pro-apoptotic factor. The absence of *COA8* (or its orthologues) was consistently associated with COX deficiency in human tissues (OMIM #616003), cells and in animal models (Yasuda et al., 2006; Sun et al., 2008; Signes et al., 2019; Brischigliaro and Zeviani, 2021).

Biallelic mutations in *COA8* were found responsible of a distinctive form of encephalopathy associated with COX deficiency (OMIM #220110) (Melchionda et al., 2014; Sharma et al., 2018; Hedberg-Oldfors et al., 2020) in 9 subjects from 8 different families (Melchionda et al., 2014; Sharma et al., 2018; Hedberg-Oldfors et al., 2020; Chapleau et al., 2023). Most of the patients reported presented progressive ataxia and spastic tetraparesis with cavitating leukodystrophy.

Here we expand the clinical and molecular findings associated with *COA8* variants by reporting two novel Italian patients presenting heterogenous clinical presentations.

## 2 Patients and methods

### 2.1 Patients

The study was approved by the institutional review board of the Fondazione IRCCS Ca’ Granda Ospedale Maggiore Policlinico. The patients provided written informed consent for all aspects of the study.

**FIGURE 1:**
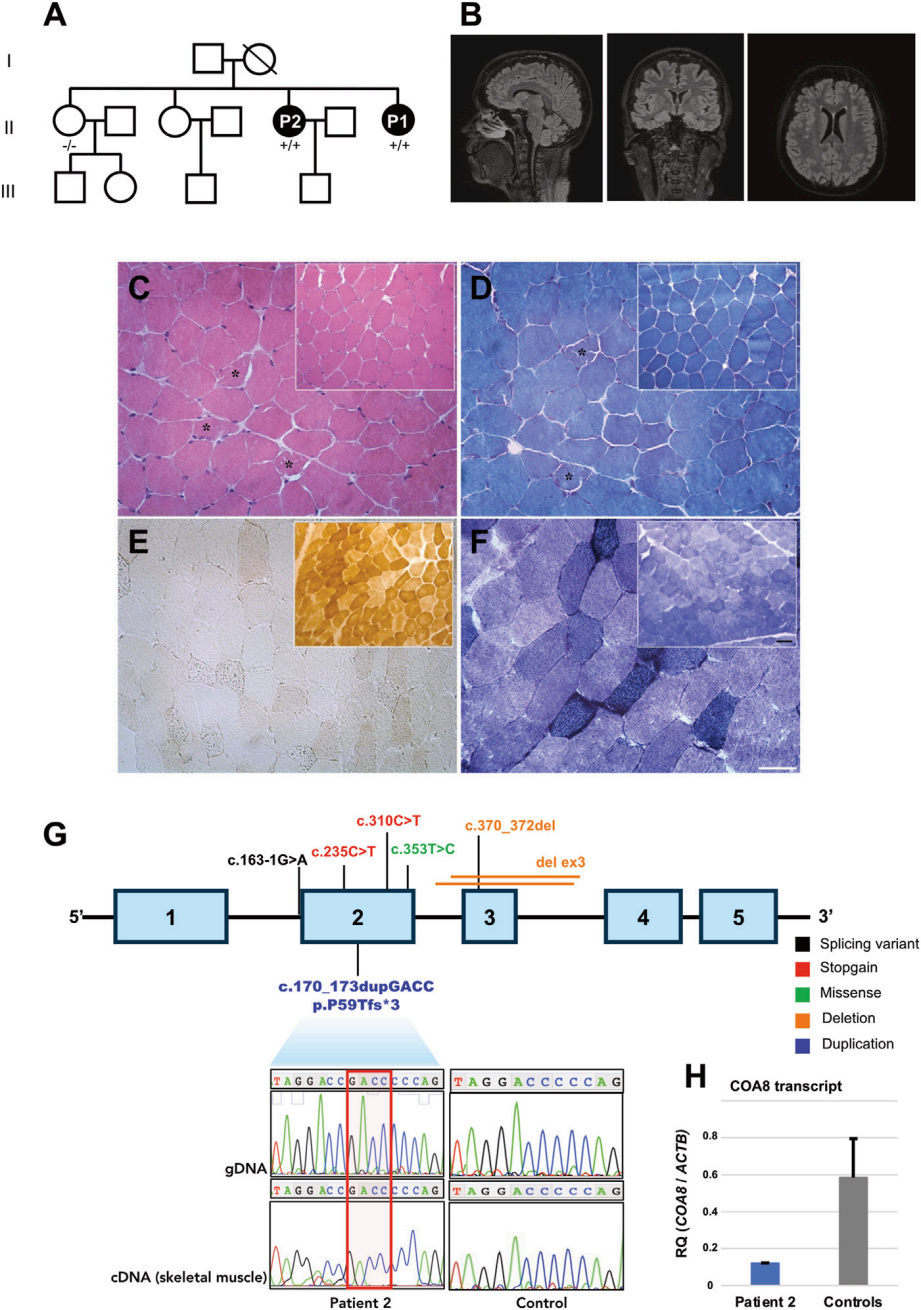
Clinical, instrumental, and genetic findings. **(A)** Pedigree and genotypes of the family investigated. Affected members are represented by black symbols (+/+: homozygous for the alternative allele; −/−: homozygous for the wild-type allele). **(B)** T2 weighted brain MRI scans of P2 showing normal findings in the sagittal (left), coronal (middle) and transverse (right) sections. **(C–F)** Histological and histochemical analysis of P2’s muscle biopsy compared to a control biopsy (inset). **(C, D)** Hematoxylin eosin (HE, **(C)** and Modified Gomori Trichrome (MGT, **(D)** staining show a preserved muscle structure. Asterisks show ragged red fibers. **(E)** Cytochrome c oxidase (COX) staining reveal a severe and diffuse reduction of COX activity in the muscle of the patient. **(F)** Succinate dehydrogenase (SDH) staining shows an increase of SDH activity in some muscle fibers. Scale bar: 50 µm. Scale bar inset: 25 µm. **(G)** Schematic representation of the *COA8* gene containing the pathogenic variants identified so far. Electropherogram showing the presence of the novel homozygous **(C)** 170_173dupGACC, p.P59Tfs*3 variant detected in genomic DNA and complementary DNA (extracted from muscle) of Patient 2. **(H)** Histograms showing relative levels of *COA8* transcript, normalized to the beta actin housekeeping gene *ACTB*, in Patient 2’s muscle compared to a group of controls (*n* = 5).

Patient 1 (P1, Figure 1A) is a 52-year-old woman, fourth-born to non-consanguineous and healthy parents of Italian origin. The prenatal and perinatal history was unremarkable. At 10 years of age, she was diagnosed with generalized epilepsy and retinitis pigmentosa. Secondary amenorrhea appeared at the age of 18.

Since her early adulthood, she complained of lower limbs exercise-induced fatigue, cramps, and myalgia.

Electromyography (EMG), performed at the age of 49 years, showed a chronic demyelinating sensorimotor polyneuropathy. Serum creatine kinase (CK) levels were normal. A muscle biopsy, performed elsewhere, had showed the presence of ragged-red fibers.

Cardiological examination revealed normal findings with an EF of 55% at the echocardiography. The last spirometry performed, at the age of 52, showed a Forced Expiratory Volume in the first second (FEV1) of 82%, slightly reduced compared to previous findings.

Brain Magnetic Resonance Imaging (MRI) was performed and showed unspecific alterations of gliotic significance, presumably due to a chronic vascular damage.

At last follow-up (52 years of age), she showed mild lower limb muscle weakness with bilateral paresthesia (“stocking” pattern). Bilateral ptosis, hypomimia, mild dysarthria and dysphonia, as well as bilateral hearing loss were observed. Walking appeared cautious, possible both on toes and heels, albeit with some difficulties. Tandem walking was impaired.

The patient currently reports muscle fatigability accompanied by cramps and pain, exacerbated after exercise.

Patient 2 (P2) is the 53-years old Patient 1’s sister.

In her early adulthood she complained of generalized asthenia, with fatigability and myalgias but never underwent medical evaluation.

At the age of 32 she developed a slowly progressive lower and upper limb muscle weakness. Premature ovarian failure at 34 years old was also reported.

The last cardiological assessment (with echocardiography and electrocardiogram, ECG) was normal except for palpitations and moderate hypertension, pharmacologically treated.

Her serum creatine kinase (CK) levels were normal or slightly elevated (last dosage at 53 years old: 110 UI/L). Serum lactate dehydrogenase (LDH) was 2.97 mmol/L, with a normal value range of 0.7–2.10 mmol/L.

At 53 years of age, a brain MRI was performed without revealing any specific alteration (Figure 1B).

A muscle biopsy, performed at the age of 32 showed the presence of ragged-red fibers and a diffuse reduction of COX activity (Figures 1C–F). Increased lipid content was noted in ragged-red fibers.

Latest neurological examination (at the age of 53 years) showed normal muscle strength, with full MRC. Mild bilateral ptosis (without ocular motility impairment) was still present.

She currently suffers from anxious-depressive syndrome and complains of vertigo with acumen, headaches, and palpitations. She also presents moderate bilateral sensorineural hearing loss at mid-high frequencies.

### 2.2 Histological and histochemical analysis

Tissue specimen was frozen in isopentane-cooled liquid nitrogen and processed according to standard techniques, as previously described. For histological analysis, 8 µm-thick cryosections were picked and processed for routine staining with Haematoxylin and Eosin (H&E), Modified Gomori Trichrome (MGT), myosin ATPase (pH 9.4-4.6-4.3), cytochrome c oxidase (COX), succinate dehydrogenase (SDH), phosphatase acid, NADH, Oil Red O, Periodic Acid Schiff (PAS). Images fields were acquired at 20X using optical microscope Leica DM4000B equipped with DFC420C camera.

### 2.3 Molecular studies

After written informed consent, genomic DNA was extracted from peripheral blood samples of proband and parents using standard procedures. The exonic regions and flanking splice junctions of the genome were captured using the Clinical Research Exome v.2 kit (Agilent Technologies, Santa Clara, CA). Sequencing was done on a NextSeq500 Illumina system with 150bp paired end reads. Reads were aligned to human genome build GRCh37/UCSC hg19.

Total RNA was extracted from Patient 2’s muscle biopsy by using Nucleozol reagent (Macherey Nagel). RNA was retrotranscribed into cDNA using Maxima Reverse Transcriptase (Thermo Scientific). RT-PCR amplification followed by sequencing was performed by using the primers: RT-F: 5′-GGA​AGA​AGA​CCT​TTC​TCC and RT-RC: 5′ GTC​AGA​GTG​GAC​TCC​TAG​TT. For quantitative experiment, we performed SYBR green qRT-PCR relative quantification analysis on a 7500 Real-Time PCR System (Applied Biosystems). The deltadeltaCt method was used to calculate the relative quantification (RQ) values of COA8 transcript (primers qRT-F: 5′- GAG​ATT​GGT​ACA​AGC​GCA​ATT​T and qRT-RC: 5′- ACT​CCT​AGT​TGC​TCC​TCT​TCT) after normalization to the housekeeping gene ACTB, encoding beta-actin (primers qRT_ACTB-F: 5′-ACG​GCT​CCG​GCA​TGT​GCA​AG and qRT_ACTB-RC: 5′-TGA​CGA​TGC​CGT​GCT​CGA​TG).

Muscle mtDNA was assayed by Southern blot and long-PCR analysis as previously described (Ronchi et al., 2012). MtDNA content and integrity were investigated by relative quantification on an ABI 7500 Real Time PCR System (Ronchi et al., 2012). MtDNA sequencing was performed by Sanger method.

### 2.4 Biochemical and protein studies

Enzymatic activities of mitochondrial respiratory chain complexes I–IV and citrate synthase (CS) were measured by spectrophotometry in the mitochondrial fractions isolated from patient’s and controls’ muscle biopsies. The specific activity of each complex was normalized to that of citrate synthase. Each experiment was performed in triplicate.

A cocktail of antibodies was used to assess mitochondrial respiratory chain subunits (Abcam ab110411, 1:1000). Additional antibodies were used for COX-I (Abcam ab14705, 1:2000), COX-III (Abcam, ab110259, 1:500), and COX-IV (Life Technologies, A21347, 1:1000) subunits. The mitochondrial PORIN (VDAC) was assayed by using a specific antibody (Abcam ab15895, 1:1500). Protein signals were detected using fluorescent secondary antibodies (LI-COR IR-DYE 800–680 CW). Actin (Sigma A2066) was used for normalization purpose.

## 3 Results

We ruled out muscle mtDNA macro rearrangements and sequence variations in Patient’s 2 muscle biopsy (haplogroup U2e1).

We performed affected-only Whole Exome Sequencing (WES) analysis and we filtered for rare variants shared by patients 1 and 2 with a coding effect compatible with a recessive inheritance. The homozygous chr14:104038005-/GACC insertion, corresponding to c.170_173dupGACC (NM_001370595, exon 2) in COA8, was prioritized (Supplementary Figure S1). This variant is expected to alter *COA8* reading frame, introducing a premature stop codon at codon 62, p.Pro59Thrfs*3 (Figure 1G).

The variant was not found in an unaffected sister, the only family members available for molecular testing. According ACMG guidelines (Richards et al., 2015) the variant satisfied the criteria PVS1 (null variant in a gene where Loss-of-function is a known mechanism of disease) and PM2 (variant not found in the Gnomad database) reaching a classification level of Likely Pathogenic. The variant was detectable in Patient 2’s muscle extracted cDNA (Figure 1G). Quantitative RT-PCR showed a moderate reduction of *COA8* transcript in Patient 2’s muscle (Figure 1H).

**FIGURE 2:**
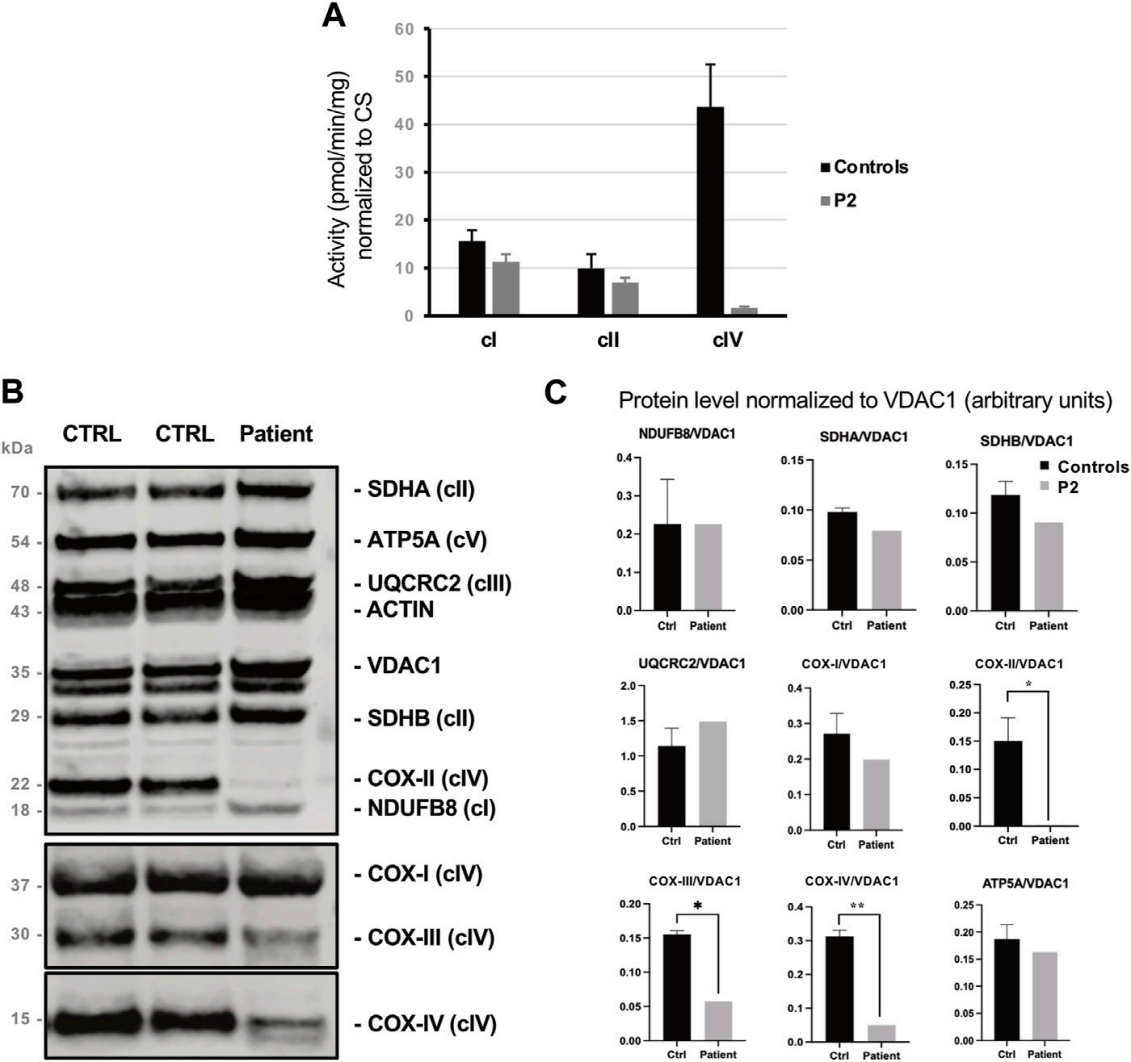
Biochemical findings collected in P2’s muscle biopsy. **(A)** Spectrophotometric analysis of respiratory chain complexes activities normalized to citrate synthase levels. Complex IV (COX) activity is reduced by >95% in P2’s muscle compared to control. **(B)** Western blot analysis of representative subunits of mitochondrial respiratory chain. **(C)** Histograms show densitometric analysis (arbitrary units) expressed as mean ± standard deviation, revealing the selective reduction of the steady state levels of Complex IV subunits COX-II, COX-III and COX-IV patient’s muscle compared to control biopsies.

Biochemical assay revealed marked increase of citrate synthase (CS) activity in muscle homogenate (patient: 329.2 pmol/min/mg; controls: 137.3 ± 15.0 pmol/min/mg), resulting in reduced values of all the respiratory chain activities when normalized to CS. In muscle, the defect in COX activity was notably more severe respect to other respiratory chain complexes (<5% of the controls’ mean, Figure 2A).

By protein analyses, we identified profound deficiency of subunits COX-II, COX-III, COX-IV and, to a lesser extent, of COX-I which are integral to the assembly of complex IV (Figures 2B, C).

## 4 Discussion

By identifying a likely causative novel pathogenic variant in a familial case of mitochondrial myopathy, this study expands the clinical and molecular features of *COA8*-related disorders. Complex IV is under a double genetic control. The three large subunits COX-I, COX-II and COX-III are encoded by mitochondrial DNA and, after local translation inside mitochondria, constitute the catalytic core of the enzyme (Fontanesi et al., 2006; Soto et al., 2012). Eleven additional subunits, with regulatory or stabilizing functions, are encoded by nuclear genes and imported into mitochondria after cytosolic translation. Finally, a set of additional proteins, known as COX auxiliary proteins, participate in the multistep pathway for maturation and assembly of the COX holocomplex. Mutations in their coding genes, all belonging to nuclear genome, result in recessively inherited clinical presentations with primary involvement of central and peripheral nervous system, skeletal and cardiac muscles (Chinnery, 1993; Zeviani and Di Donato, 2004; Russell et al., 2020). Symptoms onset usually occurs in neonatal or pediatric stage of life but growing evidence support the existence of late-onset clinical presentations (Weraarpachai et al., 2009; Richman et al., 2016; Sferruzza et al., 2021).

COA8 was initially identified as the pro-apoptotic factor APOPT1 but this function was not confirmed in further experiments. The gene is ubiquitously expressed with highest levels in skeletal muscle. The expression of COA8/APOPT1 protein, which seems actively degraded in physiological conditions, is induced by oxidative stress (Brischigliaro et al., 2019; Signes et al., 2019; Brischigliaro and Zeviani, 2021).

Its precise role in COX assembly is still unknown but it is evolutionarily conserved since the ablation of *COA8* orthologues resulted in reduction of COX activity and levels in flies and mice (Brischigliaro et al., 2019; Brischigliaro and Zeviani, 2021). These biochemical defects were also associated with a neurological phenotype in the models.

Nine patients from eight independent families have been reported harboring pathogenic *COA8* variants (Melchionda et al., 2014; Sharma et al., 2018; Hedberg-Oldfors et al., 2020; Chapleau et al., 2023) so far (Supplementary Table S1).

Symptoms onset is within 5 years of age in all the patients but one. The main clinical manifestations are encephalopathy with a peculiar posterior, supra-tentorial cavitating leukodystrophy, spastic tetraparesis, ataxia and sensorimotor polyneuropathy. Muscle weakness was reported in two patients (Melchionda et al., 2014; Hedberg-Oldfors et al., 2020). Disease course shows an early-onset rapid clinical deterioration, affecting both cognitive and motor functions over months, followed by stabilization and slow improvement over several years. Cavitating leukoencephalopathy at brain MRI was consistently observed in all the patients. An additional ninth adult case with a similar clinical presentation has been recently reported but the lack of functional validation and the benign classification of the identified missense variant question its pathogenicity (Mu et al., 2019).

In all the muscle biopsies available a diffuse reduction of histochemical COX reaction with normal SDH staining was observed. Similar findings in our patients and the absence of mtDNA defects, prompted us to look for candidate variants in nuclear DNA.

WES analysis disclosed the presence of a novel nonsense *COA8* defect. Six different mutations have been so far detected. Most of them are homozygous null variants, as those detected in our family. Other defects include a homozygous splice site mutation, resulting in the loss of a large portion of the protein, and a homozygous missense variant affecting the highly conserved Phe118 residue (Melchionda et al., 2014). No obvious genotype-phenotype correlation emerged from the reports so far published. Indeed, in our patient symptoms onset occurred during early adulthood, with lower limbs fatigability, cramps and myalgia slowly progressing to mild muscle weakness over the following two decades. Both the patients displayed mild bilateral ptosis (but not ophthalmoparesis), a symptom unreported in other patients. In addition, brain imaging in our patients did not show evidence of cavitating leukodystrophy, which was considered a neuroradiological hallmark of *COA8* mutations.

Intrafamilial clinical heterogenicity was observed in the only other reported familial case (Melchionda et al., 2014), associated with the homozygous truncating variant c.235C>T, p.(Arg79*). Indeed, one of the two *COA8*-mutated siblings had developed early-onset severe neurological impairment and became wheelchair bound at 26 years of age, while her younger sister showed a normal neurological examination at 14 years of age, although the presence of white matter abnormalities at MRI and COX deficiency in the muscle biopsy suggested a subclinical involvement.

Clinical heterogeneity was also found in our family: dysarthria and sensorimotor polyneuropathy were reported only in P1. We did not observe any further regression in our patients: the clinical course of P1 is stable while P2 showed an improvement and complete remission of muscle impairment, with almost full MRC score at the last clinical evaluation. Our two patients are 1 year apart ruling out the hypothesis of an age-related manifestation of symptoms onset.

A reduction of COX activity was observed in muscle, and to a lesser extent in primary fibroblasts of *COA8*-mutated subjects. Native experiments in immortalized fibroblasts and models also documented a severe impairment of cIV holocomplex and cIII + cIV supercomplex species which was exacerbated by induced oxidative stress. Since the stable downregulation of *COA8* mRNA in human cell lines does not produce COX biochemical impairment, these findings support the hypothesis that COA8 might protect intermediate assemblies during COX maturation from oxidant species. In our patient we also observed the selective reduction of COX subunits levels leading to a severe COX activity deficiency. Increased CS levels affected the normalized activities of other respiratory chain complexes, as previously observed in case S2 (Supplementary Table S1) of (Melchionda et al., 2014), without approaching the severity of COX activity impairment.

Mitochondrial presentation due to mutations in nuclear genes were initially suspected to display a more homogenous clinical phenotypes compared to those related to pathogenic mtDNA variants (Lax et al., 2011; Stenton and Prokisch, 2020). The increase of diagnostic yield of rare nuclear-related mitochondrial forms made possible in the last years by NGS sequencing protocols in a clinical setting challenged this idea and huge clinical variability is now commonly observed in patients presenting mutations in the same gene, even for patients with nuclear mutations. Our findings reinforce this concept also for *COA8* and prompt the consideration of this gene even in patients presenting primary mitochondrial myopathy as the preeminent clinical manifestation.

## Data Availability

The data presented in the study are deposited in the ClinVar database, accession number VCV002581720.1.
