# Supplementary material for: Prominent muscle involvement in a familial form of mitochondrial disease due to a COA8 variant
Source: Front Genet. 2023 Nov 30;14:1278572. doi: 10.3389/fgene.2023.1278572 (PMC10720436; doi:10.3389/fgene.2023.1278572)
Supplement: Supplementary file 1 [file DataSheet1.PDF]

Prominent muscle involvement in a familial form of mitochondrial disease due to a COA8 variant

Supplementary Material

A

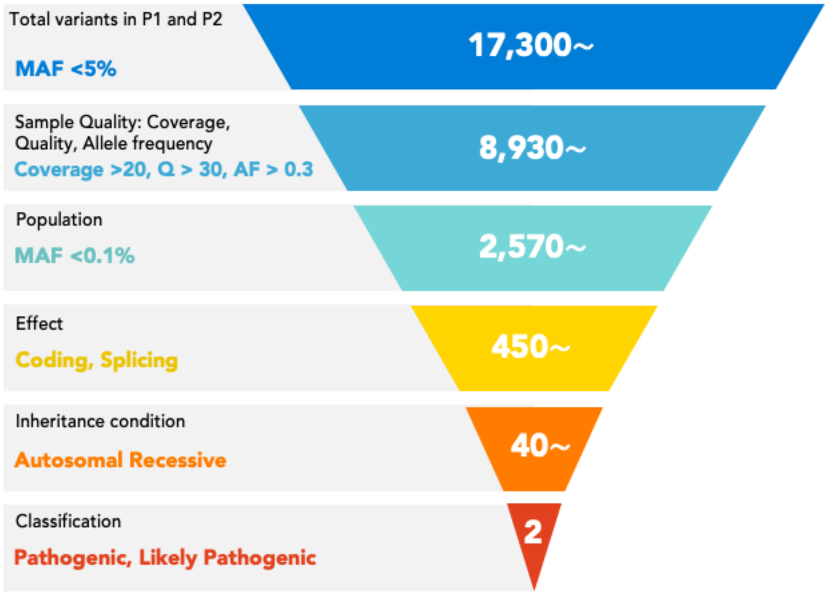

B

Chr	Position	Gene	Ref/Alt	Zygosity	cDNA	HGVS	RefSeq	ACMG Criteria
6	129722399	LAMA2	C/T	Het		R1826*	NM_000426	PVS1, PM2, PP3, PP5
**14**	**104038005**	**COA8**	**-/GACC**	**Homo**		**p.P59Tfs*3**	**NM_001370595**	**PVS1, PM2, PP3**

C

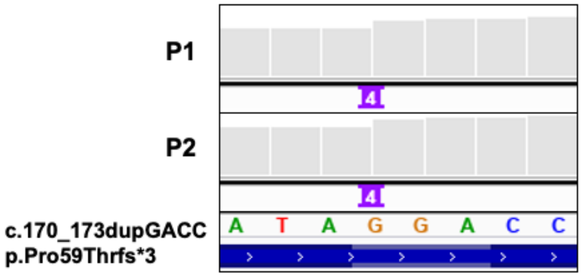

Supplementary Figure 1. (A) Prioritization workflow of NGS data. (B) Variants prioritized in the described patients. (C) IGV screenshot displaying the homozygous microduplication detected in COA8 in our patients.

Publication	Melchionda et al. 2014						Sharma et al. 2018	Hedberg-Oldfors et al. 2020	Chapleau et al., 2023	This article	
Identifier	S1	S2	S3	S4	S5	S6	Patient	Patient	Patient	P1	P2
Sex	F	F	M	M	M	F	M	F	M	F	F
Country	Italy	Italy	Turkey	Morocco	Oman	Italy	India	Sweden	Unknown	Italy	Italy
Initial motor & cognitive development	Normal	Normal	Normal	Normal	Mildly delayed	Normal	Normal	Normal	Normal	Normal	Normal
Age at presentation (years)	2.5	Never developed neurological signs	3	5	5	2	5	3	3.5	10	32
Initial symptoms	Hemiparesis, somnolence, loss of ambulation	NA	Delayed speech, gait difficulties	Gait difficulties	Delayed psychomotor development dysarthria and gait difficulties	Frequent falls and leg weakness	Subacute onset of neuro-regression with right-sided focal seizures	Muscle weakness, dysphagia, dysarthria, and gait ataxia	Acute motor regression	Lower limbs exercise-induced fatigue, cramps, and myalgia. Secondary amenorrhea (18 years)	Asthenia, fatigability, and myalgia. POF (34 years)
Regression symptoms (duration)	Severe spastic tetraparesis, lowered consciousness (2 months)	NA	Spastic tetraparesis left>right (2 years)	Spastic tetraparesis, ataxia and sensorimotor polyneuropathy with loss of unsupported walking (2–3 months)	Spastic tetraparesis, ataxia and sensorimotor polyneuropathy with loss of unsupported walking (2–3 months)	Spastic tetraparesis and sensorimotor polyneuropathy with loss of ambulation; gastrostomy due to swallowing defect (5 months)	Loss of unsupported walking and sitting; loss of speech; severe limbs stiffness; loss of hearing and sight (deceased after 6 months for aspiration pneumonia)	Loss of ambulation and speech, dystonia, spasticity, and gastrostomy due to dysphagia (2.5 years)	Severe spasticity, truncal hypotonia, left hemiparesis, loss of ambulation, cognitive and speech regression (3 weeks).	mild lower limb muscle weakness with bilateral paresthesia, exercise-induced muscle fatigability, cramps with pain exacerbating after long walks; sensorimotor polyneuropathy; bilateral ptosis, hypomimia; dysphonia; bilateral hearing loss (>20 years)	4 limbs muscle weakness, difficulties walking; bilateral ptosis; bilateral hearing loss (>15 years)
Follow up	Seizures at the age of 4 years, no further regression	NA	Episodes of seizures, no further regression	No further regression	No further regression	No further regression	NA	Slowly improved, regaining most of the lost functions	Further regression (after 2 weeks of stabilization): seizures, dysautonomia, encephalopathy (2 months)	No further regression (stabilization)	No further regression, slowly improved regaining motor functions
Age at last examination (years)	26	14	16	13	6.5	4	NA	25	4 (death)	52	53
Motor function	Wheelchair-bound	Normal	Moderate spastic tetraparesis left > right; wheelchair-bound	Walks, mild signs of spasticity, ataxia, and peripheral neuropathy	Walks, mild signs of spasticity, ataxia, and peripheral neuropathy	Walks, spastic gait	NA	Walks, spastic gait, and ataxia	Loss of ambulation	Walks (with difficulties)	Walks, normal muscle strength
Cognitive level	Decreased	Normal	Decreased	Slightly decreased	Normal	Normal	NA	Slightly decreased, lives independently, and works part time	Decreased	Normal	Normal
Speech and language	Single words, marked dysarthria	Normal	Dysarthria	Normal	Normal	Normal	NA	NA	Dysarthria, slowed speech	Mild dysarthria; dysphonia	Normal
Biochemical analysis	COX deficiency	COX deficiency	COX deficiency	COX deficiency	NA	COX deficiency	COX deficiency	COX deficiency	NA	NA	Reduced respiratory chain activities, in particular COX. Decreased levels of COX-II, COX-III, COX-IV and COX-I proteins.

Muscle pathology at light microscopy	Reduced COX and SDH normal	Reduced COX and SDH normal	Reduced COX and SDH normal	NA	NA	Reduced COX and SDH normal	Reduced COX	Reduced COX	NA	Ragged-red fibers	Diffuse reduction of COX activity. SDH activity preserved. Ragged-red fibers with increased lipid content
Electron Microscopy	Mitochondria with osmiophilic inclusions and disorganization of the cristae		NA	NA	NA	NA	NA	Enlarged mitochondria and slight lipid accumulation	NA	NA	NA
Brain defects/MRI	Severe reduction of the entire WM with multiple small cysts and enlarged lateral ventricles (21 year)	Abnormal signal and small cysts in the parietooccipital WM and corpus callosum (15 years)	Abnormal signal and numerous small cysts in the parieto-occipital white matter and corpus callosum. Small lesions in the frontal and temporal WM (4 years)	Abnormal signal and numerous small cysts in the parieto-occipital white matter and corpus callosum. Small lesions in the frontal WM (5 years)	Abnormal signal and numerous small cysts in the parieto-occipital white matter and corpus callosum. Small lesions in the frontal and temporal WM (5 years)	Abnormal signal and numerous small cysts in the parieto-occipital white matter and corpus callosum. Small lesions in the frontal and temporal WM (3 years)	Diffuse WM abnormalities and multiple cysts with posterior predominance (5 years)	Abnormal signal with cystic lesions mainly in the parieto-occipital-temporal WM (3 years)	Bilateral symmetric cavitating leukodystrophy. Mild atrophy (3.5 years)	MRI: unspecific alterations of gliotic significance (chronic vascular damage) (52 years)	MRI: normal signal with minimal unspecific alterations (53 years)
DNA	c.235C>T	c.235C>T	c.163-1G>A	Ex3 deletion	c.353T>C	c.235C>T; c.370_372del	Ex3 deletion	c.310C>T	Ex3 deletion	c.170_173dupGACC	c.170_173dupGACC
Protein	p.Arg79*	p.Arg79*	Ex2 skipping; p.Val55_Lys120del	p.Glu121Valfs*4	p.Phe118Ser	p.Arg79*; p.Glu124del	p.Glu121Valfs*4	p.Q104*	p.Glu121Valfs*4	p.P59Tfs*3	p.P59Tfs*3
Genotype	Homoz	Homoz	Homoz	Homoz	Homoz	Compound heterozygous	Homoz	Homoz	Homoz	Homoz	Homoz

Supplementary Table 1 Clinical and molecular features of the *COA8*-mutated patients so far reported.
Abbreviations: NA = Not Available; Homoz: Homozygous; WM = White Matter; COX = Cytochrome c Oxidase; SDH = Succinate Dehydrogenase; Ex = Exon; POF = Premature Ovarian Failure.
